# Relationships between cognition, functioning, and quality of life of euthymic patients with bipolar disorder: Structural equation modeling with the FACE-BD cohort

**DOI:** 10.1192/j.eurpsy.2024.1789

**Published:** 2024-11-15

**Authors:** P Roux, S Frileux, N Vidal, V Aubin, R Belzeaux, P Courtet, C Dubertret, B Etain, E Haffen, M Leboyer, A Lefrere, PM Llorca, K M’Bailara, E Marlinge, E Olié, M Polosan, R Schwan, The FACE-BD (FondaMental Academic Centers of Expertise for Bipolar Disorders) group, E Brunet-Gouet, C Passerieux

**Affiliations:** 1 Fondation FondaMental, Créteil, France; 2 Centre Hospitalier de Versailles, Service Universitaire de Psychiatrie d’Adultes et d’Addictologie, Le Chesnay; Université Paris-Saclay; Université de Versailles Saint-Quentin-En-Yvelines; DisAP-DevPsy-CESP, INSERM UMR1018, Villejuif, France; 3 Pôle de Psychiatrie, Centre Hospitalier Princesse Grace, Av. Pasteur, Monaco; 4 Pôle universitaire de psychiatrie, CHU de Montpellier, Montpellier, France; 5 Department of Emergency Psychiatry and Acute Care, CHU Montpellier, IGF, Univ. Montpellier, CNRS, INSERM, Montpellier, France; 6 AP-HP, Groupe Hospitalo-Universitaire AP-HP Nord, DMU ESPRIT, Service de Psychiatrie et Addictologie. Hopital Louis Mourier, Colombes, Inserm U1266, Faculté de Médecine, Université Paris Cité, France; 7 AP-HP, Groupe Hospitalo-universitaire AP-HP Nord, DMU Neurosciences, Hôpital Fernand Widal, Département de Psychiatrie et de Médecine Addictologique, Paris, France; Université Paris Cité, INSERM UMR-S 1144, Optimisation Thérapeutique en Neuropsychopharmacologie OTeN, Paris, France; 8 Université de Franche-Comté, UR LINC, Département de Psychiatrie Clinique, CIC-1431 INSERM, CHU de Besançon, 25000 Besançon, France; 9 Univ Paris Est Créteil, INSERM U955, IMRB, Translational NeuroPsychiatry Laboratory, Créteil, France; AP-HP, Hôpitaux Universitaires Henri Mondor, Département Médico-Universitaire de Psychiatrie et d’Addictologie (DMU IMPACT), Fédération Hospitalo-Universitaire de Médecine de Précision en Psychiatrie (FHU ADAPT), Créteil, France; 10 Pôle de Psychiatrie, Assistance Publique Hôpitaux de Marseille, Marseille, France; INT-UMR7289, CNRS Aix-Marseille Université, Marseille, France; 11 Centre Hospitalier et Universitaire, Département de Psychiatrie, Clermont-Ferrand, France; Université d’Auvergne, EA 7280, 63000 Clermont-Ferrand, France; 12 Centre Hospitalier Charles Perrens, Pôle PGU; LabPsy, UR 4139 Université de Bordeaux, Bordeaux, France.; 13 Université Grenoble Alpes, CHU de Grenoble et des Alpes, Grenoble Institut des Neurosciences (GIN) Inserm U 1216, Grenoble, France; 14 Université de Lorraine, Centre Psychothérapique de Nancy, Inserm U1254, Nancy, France

**Keywords:** antipsychotic, anxiety, bipolar disorders, cognition, depression, psychosis, quality of life

## Abstract

**Background:**

Quality of life is decreased in bipolar disorders (BD) and contributes to poor prognosis. However, little is known about the causal pathways that may affect it. This study aimed to explore health-related QoL (HRQoL) in BD and investigate its relationship with cognition and psychosocial functioning.

**Methods:**

This multicenter cross-sectional study used a neuropsychological battery to assess five cognition domains. Functioning was evaluated using global and domain-based tools, and health-related HRQoL was assessed using the EQ-5D-3L. Structural equation modeling was used to test whether the association between cognition and HRQoL would be mediated by functioning in BD while controlling for covariates such as residual depression, anxiety, antipsychotic medication, and psychotic features.

**Results:**

We included 1 190 adults with euthymic BD. The model provided a good fit for the data. In this model, the direct effect of cognition on HRQoL was not significant (β = − 0.03, *z =* −0.78, *p =* 0.433). The total effect of cognition on HRQoL was weak, albeit significant (β = 0.05, *z =* 3.6, *p <* 0.001), thus suggesting that cognition affected HRQoL only indirectly through functioning. Anxiety was associated with decreased functioning (β = −0.27, *z =* −7.4, *p <* 0.001) and QoL (β = −0.39, *z =* −11.8, *p <* 0.001).

**Conclusions:**

These findings suggest that improving cognition may not directly lead to a higher HRQoL. Cognitive remediation is expected to improve HRQoL only through functioning enhancement. They also reveal the potential importance of functional remediation and reduction of comorbid anxiety symptoms in improving HRQoL in BD.

## Introduction

Quality of life (QoL) is a broad construct defined as satisfaction in the physical, psychological, social, and environmental aspects of life, which is significantly lower for individuals with BD in remission than those without lifetime BD [1]. BD is associated with a reduction in the self-reported QoL to a similar [2] or even greater extent than schizophrenia [3]. Among the several dimensions of QoL, health-related QoL (HRQoL) refers to the individual’s perception of physical and mental health over time and its impact on the ability to live a fulfilling life. HRQoL is particularly low in BD [4].

Several studies have reported positive associations between QoL and neuropsychological performance in executive functioning and verbal abstraction [5–7]. This association may be particularly present following remission of the first manic episode [8]. Another study involving 224 participants reported that cognitive reserve was positively associated with the physical component of HRQoL but negatively associated with its mental component [9]. The association between good neuropsychological performance and a satisfying QoL thus needs to be confirmed in studies with large sample sizes.

Moreover, the studies investigating associations between cognition and QoL did not consider the potential mediating role of functioning. Condition-specific functioning measures [10] and psychological functioning [11] are indeed positively associated with QoL in BD. When measured with BD-specific tools, QoL is also positively associated with functioning [12]. Cognition is a critical determinant of functioning in BD: a study using elastic net regression reported that executive functioning was associated with functioning (β = 0.30) [13]. A previous SEM study reported a significant association (β = 0.18) between cognition and functioning when controlling for premorbid IQ, number of episodes, and lithium response [14]. This association was confirmed with longitudinal studies: processing speed predicts long-term functioning [15] and verbal memory middle-term functioning [16]. It is thus crucial to investigate whether the positive association sometimes found between cognition and QoL is mediated by functioning.

Previous studies investigating the association between cognition and QoL have also ignored essential confounders. Several determinants of cognition, functioning, and QoL have been identified in BD. First, residual depressive symptoms are associated with QoL [17], cognition [18], and functioning [19] in individuals with BD in euthymia. Subsyndromal anxiety has also been identified as an important predictor of poor QoL in BD [20] and comorbid anxiety is associated with poor neuropsychological performance in BD-II [21]. Moreover, a history of psychosis in BD is associated with poorer cognition [22]. Finally, medication is associated with QoL in BD through adverse effects [2]. The prescription of antipsychotics has also been shown to be associated with cognitive impairment in a meta-analysis of cross-sectional studies, including euthymic participants with BD [23]. It is, thus, likely of particular importance to control for antipsychotic prescription when investigating the relationship between cognition, functioning, and QoL in BD.

This study aimed to explore the relationship between cognition, functioning, and HRQoL in a large sample of individuals with euthymic BD using SEM and controlling for several covariates, such as subthreshold depression, anxiety, psychotic features, and antipsychotic medication. We hypothesized that the positive association between cognition and HRQoL would be mediated by functioning and that these covariates would not confound this mediation.

## Methods

The present report followed the Strengthening the Reporting of Observational Studies in Epidemiology (STROBE) guidelines.

### Study design and characteristics of the recruiting network

This multicenter transversal study included patients recruited into the FACE-BD (FondaMental Advanced Centers of Expertise for Bipolar Disorders) cohort within a French national network of 10 centers (Bordeaux, Colombes, Créteil, Grenoble, Marseille, Monaco, Montpellier, Nancy, Paris, and Versailles). All procedures contributing to this work were approved by the local ethics committee (*Comité de Protection des Personnes Ile de France IX*) on January 18, 2010.

### Participants

The diagnosis of BD was based on the Structured Clinical Interview for DSM-IV-TR (SCID) criteria [24]. Outpatients with type 1, type 2, or not-otherwise-specified (NOS) BD between 18 and 65 years of age were eligible for this analysis. All patients included in the analyses were euthymic at the time of testing according to the DSM-IV-TR criteria, with scores on the Montgomery-Asberg Depression Rating Scale (MADRS) ≤ 11 [25] and the Young Mania Rating Scale (YMRS) < 12 [26]. Patients who met the following criteria at any time of testing were excluded to control for a cognitive impairment resulting from a cause other than BD itself: history of significant neurological disorder, dyslexia, dysorthographia, dyscalculia, dysphasia, dyspraxia, substance-related disorders in the previous month (except tobacco use), electroconvulsive therapy in the past year. Patients with anomalous trichromatism and any disabling visual impairment were also excluded to avoid an artefactual cognitive impairment.

### Assessment tools

#### Clinical evaluation

The subtype of BD and history of psychotic features were established according to DSM-IV-TR criteria. The MADRS total score measured residual depressive symptoms. The presence of antipsychotics during the time of testing was reported as a binary variable. The state of anxiety was measured using the state subscale of the State–Trait Anxiety Inventory, form Y-A (STAI-Y-A) [27].

#### Cognition

The neuropsychological battery explored five cognitive domains through 10 tests, five of which were subtests from the WAIS version III [28] or version IV [29], as the French version of the WAIS-IV started to be used as it became available.

- Verbal memory: California Verbal Learning Test [30] short- and long-delay free recall
- Working memory: WAIS digit span (total score) and spatial span (forward and backward scores) from the Wechsler Memory Scale version III [31]
- Executive functions: Trail Making Test (TMT) part B [32], color/word condition of the Stroop test [33], semantic and phonemic verbal fluency [34]
- Processing speed: Digit symbol coding (WAIS-III) or coding (WAIS-IV), WAIS symbol search, and TMT part A
- Verbal and perceptual reasoning: WAIS vocabulary and matrices

Raw scores were transformed into demographically corrected standardized z-scores based on normative data [33, 35–37]. Higher scores reflect better performance. We computed a mean score for each cognitive domain.

#### Functioning

Global functioning was measured using the Global Assessment of Functioning (GAF) [38]. Domain-based psychosocial functioning in everyday life was assessed using the Functioning Assessment Short Test (FAST) [39]. For the GAF, higher scores are associated with higher functioning, whereas the inverse is true for the FAST.

#### HRQoL

HRQoL was measured with the European Quality of Life 5 Dimensions and 3 Lines (EQ-5D-3L) value index. It is a generic preference-based measure developed to describe and value health across various disease areas [40]. The scale evaluates five aspects of health: mobility, self-care, usual activities, pain/discomfort, and anxiety/depression. Each dimension has three levels: no, some, and extreme. EQ-5D health states were converted into a single summary number, the index value, using a time trade-off valuation technique [41]. An index value of 1 corresponds to the best possible health state according to the preferences of the general population of a country/region, while an index value of <0 represents the worst possible health state. It has shown good convergent validity [42, 43] and known-groups validity in patients with and without BD [43–45].

### Statistical analyses

#### Rationale to map the latent variables with their indicators

The five average scores for the different cognitive domains were included in the SEM model as indicators of the latent variable cognition. They all corresponded to theoretically separated, albeit partially overlapping, cognitive functions. Factor analyses previously identified working memory [46], conceptual reasoning [47], psychomotor speed, verbal memory, and executive functioning [48] as autonomous cognitive dimensions in BD.

Two measures were included in the SEM model as indicators for the latent variable functioning: GAF & FAST total score. They were chosen because they measured complementary aspects of functioning. GAF assesses how much a person’s symptoms affect day-to-day life. In contrast, the FAST-total score combines specific domains of functioning (autonomy, occupational functioning, cognition, financial issues, interpersonal relationships, and leisure), irrespective of the symptoms’ level. Several studies have reported that these two types of functioning assessments are closely associated in BD [49, 50].

#### Rationale for covariates selection

Four covariates were included in the SEM model because they have all been associated with cognitive deficits in BD: prescription of antipsychotics [23], psychotic features [22], residual depressive symptoms [18], and anxiety [21].

#### Model specifications, estimation, and testing

Zero-order correlations between each measure (cognition, functioning, HRQoL, and covariates) were calculated using Spearman’s correlation coefficients.

Linear regression analyses evaluated relationships between cognition, functioning, and HRQoL. They were indexed using standardized coefficients: factor loading for the relationship between indicators and latent variables and standardized regression coefficients (β) for the relationship between covariates, cognition, functioning, and HRQoL. Two cognitive domain scores were computed from two cognitive variables acquired during the same cognitive test (the TMT, in which part A was included in speed of processing, whereas part B was included in executive functions), leading to a shared method variance. To account for the artefactual influence of the current assessment method, the residual variances between the implied indicators were allowed to be correlated, as recommended [51].

A robust estimator was used: the maximum likelihood estimation with robust (Huber-White) standard errors and a scaled test statistic (asymptotically) equal to the Yuan-Bentler test statistic. Missing data were managed using the full information maximum likelihood (FIML). We examined consensual fit indices with recommended cutoff criteria for good fit [52]: Comparative Fit Index (CFI) and Tucker-Lewis Index (TLI) > 0.95, root mean square error of approximation (RMSEA) ≤ 0.05 (p of close-fit >0.05, and 90% confidence intervals), standardized root mean square residual (SRMR) < 0.08. For the mediation analysis, the direct effect was the standardized path coefficient between cognition and functioning, and the indirect effect was the product of the direct effect and the standardized path coefficient between functioning and HRQoL. The total effect of cognition on HRQoL was the sum of direct and indirect effects.

## Results

### Characteristics of the participants

**Figure 1.**
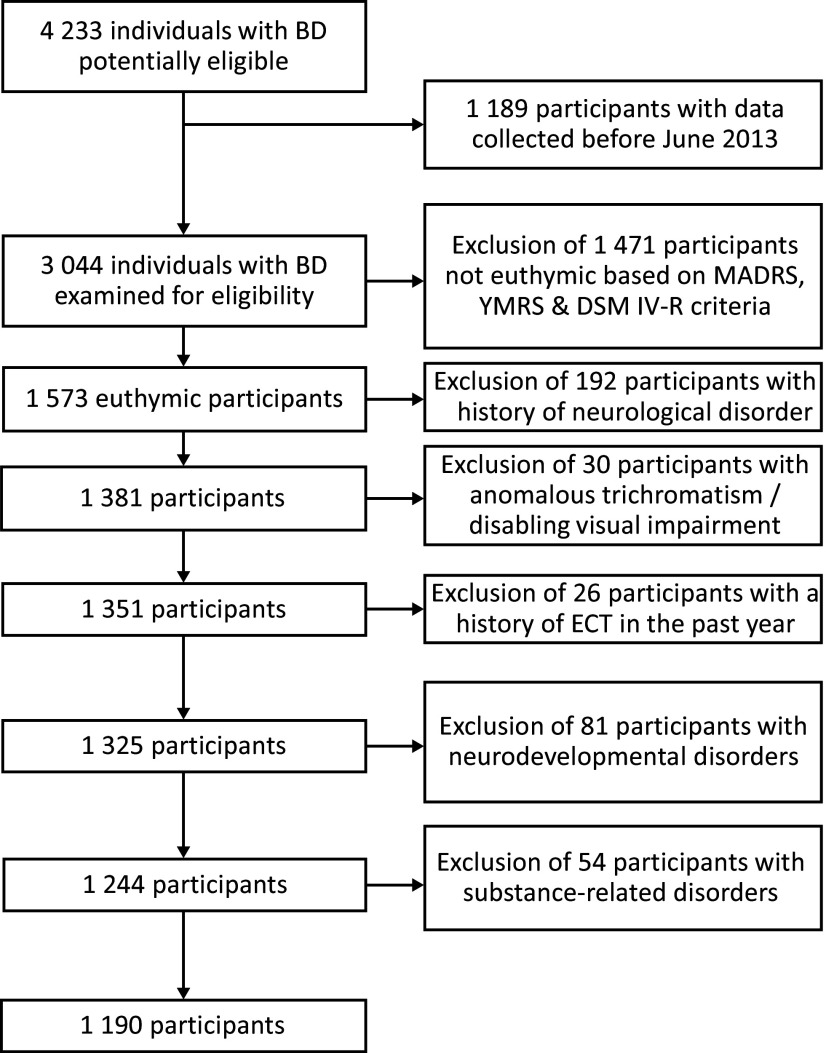
Flowchart of the study participants’ selection process.

The flowchart of the study participants’ selection process is reported in Figure 1. The inclusion period began in June 2013, when HRQoL started to be routinely collected by all centers, and ended in January 2021. We confirmed the eligibility for 1190 participants whose data were then analyzed. The participants’ sociodemographic, clinical, and functional characteristics are reported in Table 1. The participants mainly consisted of women with type 1 BD.

**Table 1.** Participant socio-demographic, clinical, and functional characteristics

Variable	Mean or %	SD	*N* missing
Age (years)	38.7	12.4	0
Sex	35.9 (men)		2
Education level (years)	14.5	2.6	78
Diagnosis	50.9 Type I		0
	40.4 Type II		
	8.7 NOS		
MADRS total score	4	3.2	49
YMRS total score	1.6	2.5	50
Psychotic features	0.4	0.5	234
Antipsychotics	42.7 (present)		288
STAI-YA, state subscore	35.9	12	95
FAST total score	14.2	11.1	58
GAF	73.1	12.6	194
EQ–5D–3L	0.8	0.2	258

Abbreviations: N missing: Number of participants with missing information; MADRS: Montgomery-Asberg Depression Rating Scale; YMRS: Young Mania Rating Scale; STAI-YA: State–Trait Anxiety Inventory, form Y-A; FAST: Functioning Assessment Short Test; GAF: Global Assessment of Functioning; EQ-5D-3L: European Quality of Life 5 dimensions and 3 lines.

The neuropsychological results are presented in Table 2. WAIS-III was proposed to 60 participants and WAIS-IV to 927 participants. The worst performances were found for verbal memory (−0.07 ± 1.11) and executive functioning (−0.05 ± 0.73), and the best for reasoning (0.21 ± 0.82).

**Table 2.** Cognitive performance expressed in demographically corrected standardized z-scores

Variable	Mean	Sd	*N* missing
**Verbal memory**	−0.07	1.11	253
CVLT Short delay-free recall	−0.09	1.13	253
CVLT Long delay-free recall	−0.06	1.16	253
**Working memory**	−0.03	0.72	230
Digit Span	−0.04	0.94	238
Spatial Span forward	−0.01	0.89	282
Spatial Span backward	−0.06	0.85	282
**Executive functioning**	−0.05	0.73	230
TMT Part B	−0.15	1.01	240
Stroop color/word condition	0.05	1.03	241
Phonemic fluency	0.06	1.09	244
Semantic fluency	−0.15	1.02	246
**Processing speed**	0.03	0.71	227
Digit symbol coding / Coding	−0.14	0.94	232
Symbol search	0.07	0.91	233
TMT Part A	0.17	0.81	239
**Reasoning**	0.21	0.82	245
Vocabulary	0.64	1.05	533
Matrices	−0.03	0.88	249

Abbreviations: CVLT: California Verbal Learning Test; TMT: Trail Making Test; CPT: Continuous Performance Test.

The mean FAST total score was 14.2 (± 11.1), and the mean GAF score was 73.1 (± 12.6), corresponding to mild functional impairment [49].

The mean EQ-5D-3L index was 0.831 (± 0.178), which was below the scores reported for different age classes below 65 in the general French population (ranging from 0.948 in the 18–24 year class to 0.853 in the 55–64 year class) [53].

### SEM model

The zero-order correlations matrix is reported in Supplementary Table 1. There were 14.8% missing data with 99 different patterns of missingness. The covariance coverage matrix of missing data is reported in Supplementary Table 2. The observed variances for EGF, FAST, & STA-Y (state subscale) were at least 1000 times greater than the observed variance of some other variables. They were thus converted to z-scores. The model provided a good fit for the data, as suggested by TLI and CFI above 0.95 (TLI = 0.97, CFI = 0.982), RMSEA not statistically greater than 0.05 (RMSEA = 0.031, one-sided *p*-value of the test of the null hypothesis that the RMSEA equals .05: 1) and an SRMR below 0.08 (0.026). All indicator variables were reliable and valid measures of their respective latent variables, as supported by significant moderate to high factor loadings (absolute values of standardized factor loadings ≥0.5, *p* < 0.001, see Supplementary Figure 1 and Table 3). The model explained 36.3% of the variance in functioning and 37.4% of the variance in HRQoL.

**Table 3.** Statistics for the estimated factor loadings, standardized path coefficients, and correlation coefficients of the SEM mediation model

Variables	Standardized coefficient	Standard Error	z	p
**Latent variables**				
Cognition → verbal memory	0.498	0.03	16.7	<0.001
Cognition → working memory	0.656	0.03	21.9	<0.001
Cognition → executive functions	0.71	0.029	24.8	<0.001
Cognition → speed processing	0.639	0.031	20.5	<0.001
Cognition → reasoning	0.519	0.035	14.8	<0.001
Functioning → GAF	0.718	0.028	25.9	<0.001
Functioning → FAST	−0.798	0.026	−31.2	<0.001
**Regressions**				
Cognition ← MADRS	−0.055	0.041	−1.3	0.179
Cognition ← STAI-YA (state subscale)	−0.073	0.042	−1.8	0.079
Cognition ← antipsychotics	−0.227	0.04	−5.7	<0.001
Cognition ← psychotic features	−0.102	0.041	−2.5	0.013
Functioning ← MADRS	−0.35	0.037	−9.5	<0.001
Functioning ← STAI-YA (state subscale)	−0.273	0.037	−7.4	<0.001
Functioning ← antipsychotics	−0.072	0.038	−1.9	0.058
Functioning ← psychotic features	−0.002	0.035	−0.1	0.945
Functioning ← cognition	0.205	0.041	5	<0.001
EQ–5D–3L ← MADRS	−0.088	0.037	−2.4	0.017
EQ–5D–3L ← STAI-YA (state subscale)	−0.393	0.033	−11.8	<0.001
EQ–5D–3L ← antipsychotics	−0.005	0.033	−0.1	0.885
EQ–5D–3L ← psychotic features	0.069	0.031	2.2	0.026
EQ–5D–3L ← cognition	−0.03	0.039	−0.8	0.434
EQ–5D–3L ← functioning	0.254	0.047	5.4	<0.001
**Covariances**	0.335	0.045	7.4	<0.001
Executive functions ↔ speed processing	−0.055	0.041	−1.3	0.179

Abbreviation: GAF: Global Assessment of Functioning; FAST: Functioning Assessment Short Test; MADRS: Montgomery-Asberg Depression Rating Scale; STAI-YA: State–Trait Anxiety Inventory, form Y-A; EQ-5D-3L: European. Quality of Life 5 dimensions and 3 lines.

**Figure 2.**
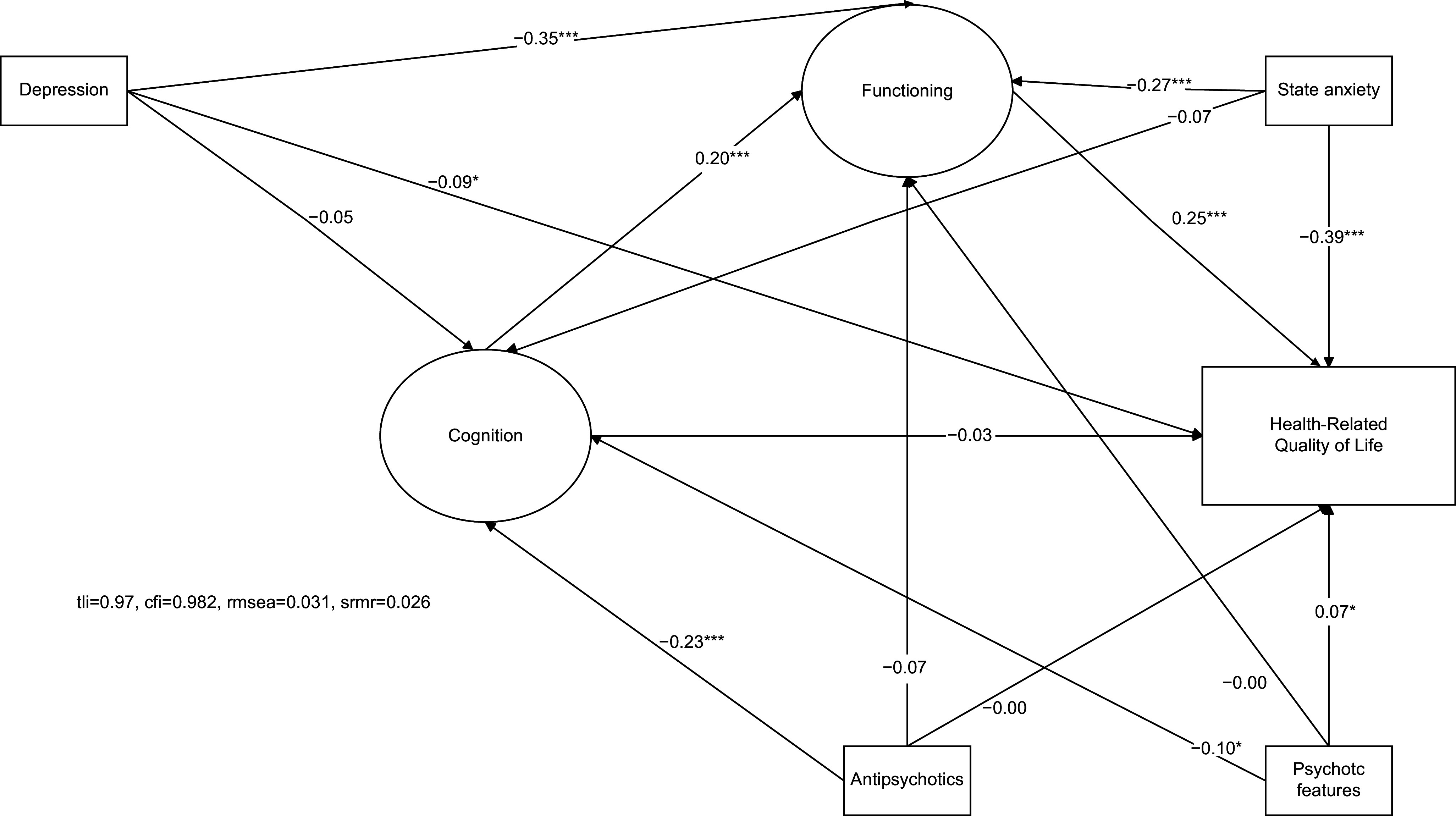
Simplified diagram of the model. Indicators of latent variables were omitted for readability (see Supplementary Figure 1 to see the model with the indicators). Rectangles indicate the observed variables, ovals the latent variables, single-headed arrows the regressions (freely estimated regression weight), and double-headed arrows the covariances. Path coefficients were standardized. (Significance levels are as follows: ***p < 0.001, **p < 0.01, *p < 0.05).

The association between cognition and HRQoL was not significant (see Figure 2), whereas cognition was significantly and positively associated with functioning (β = 0.20, *z =* 5, *p <* 0.001), and functioning was significantly and positively associated with HRQoL (β = 0.25, *z =* 5.4, *p <* 0.001). The mediation analysis showed a nonsignificant direct effect of cognition on HRQoL (β = − 0.03, *z =* −0.78, *p =* 0.433), a significant indirect effect of cognition on HRQoL (β = 0.05, *z =* 3.6, *p <* 0.001) and a non-significant total effect of cognition on HRQoL (β = 0.02, *z =* 0.56, *p =* 0.567). The significant indirect effect in the absence of a significant direct effect was interpreted as a full mediation (or indirect-only mediation) of the effect of cognition on HRQoL by functioning. The significant indirect effect in the absence of a significant total effect of cognition on HRQoL was interpreted as an inconsistent mediation: the positive indirect effect of cognition on HRQoL was neutralized by its negative direct effect.

Antipsychotic prescription negatively affected cognition (β = −0.23, *z =* −5.7, *p <* 0.001, see Figure 2 and Table 3). Two covariates were significantly and negatively associated with functioning: depression (β = −0.35, *z =* −9.5, *p <* 0.001) and state anxiety (β = −0.27, *z =* −7.4, *p <* 0.001). Beyond functioning, HRQoL was mainly negatively associated with state anxiety (β = −0.39, *z =* −11.8, *p <* 0.001).

## Discussion

The present study aimed to investigate the relationships between cognition, functioning, and HRQoL in a large sample of individuals with euthymic BD, controlling for a set of covariates.

The main result of this study is that cognition was only indirectly associated with HRQoL in euthymic participants with BD: better cognition was associated with better psychosocial functioning, which was, in turn, associated with better HRQoL. There was no direct link between cognition and HRQoL, thus confirming previous findings in more symptomatic participants with BD, controlling for cognitive reserve [54]. One explanation for this lack of a direct association could have been the use of a global measure of QoL, which may combine subdimensions with opposite associations with cognition. For example, a previous study reported a negative association between cognitive reserve and the psychological dimension of HRQoL, whereas its physical dimension was positively associated with cognitive reserve [9]. Therefore, further studies are encouraged using more specific measures of QoL when investigating its association with cognition in BD. This result has an important impact on recommendations about cognitive remediation in BD. Clinicians should expect an improvement in HRQoL after cognitive remediation for individuals with BD and cognitive impairment through functioning enhancement. A recent RCT investigated the transfer of cognitive remediation into functioning and goal attainment, with mixed findings [55]. First, improvement in cognition accounts for more than one-third of the cognitive remediation effect on functioning. Second, cognitive improvements did not account for changes in goal attainment, a key component of QoL. However, the cognitive level achieved following remediation accounts for differences in goal attainment since only those performing above a certain level showed significant improvement in goal attainment. It is important to note that the comparison between the two studies is limited by the fact that the goal attainment measured in this study was quite different from the HRQoL measured in ours. Another RCT reported a positive effect of cognitive remediation on executive function in BD but a lack of improvement of functioning and QoL, thus suggesting again that cognitive improvement without functional improvement is insufficient to enhance QoL [56]. An inconsistent mediation of the effect of cognition on QoL by functioning has been previously identified in schizophrenia [57]. The neutralization of the positive indirect effect of cognition on HRQoL by its negative direct effect may be explained by the fact that individuals with better cognition, resulting from a higher cognitive reserve, may have an increased awareness of their disorder and thus overestimate its consequences compared to their premorbid situation, leading to a lower perceived HRQoL. Further studies should control from clinical insight when investigating the associations between cognition, functioning, and QoL in BD.

Several modifiable factors of HRQoL were identified in our study, suggesting certain amendments to the classification of QoL determinants in BD between marked, moderate, mild, possible, and unknown proposed by Grunze and Born [58]. Our results confirm the marked impact of anxiety on decreased QoL reported in this review. They contrast those in a previous study showing the lack of an association between anxiety and QoL in a sample of individuals with BD when residual depressive symptoms were accounted for [17]. Several approaches have been proposed to relieve anxiety in BD, such as mindfulness-based cognitive therapy [59] and medication optimization [60, 61]. Our results suggest these approaches may also improve functioning and HRQoL in BD at euthymia. Functioning should also be added to the category of marked moderators of HRQoL in BD. Functional remediation, which aims to develop cognitive strategies, psychoeducation about cognition, and problem-solving in the context of everyday life improves the functional outcome in euthymic individuals with BD [62, 63]. Our results thus suggest that this intervention may also improve HRQoL in BD. Potential concerns have been raised toward the use of the EQ-5D in BD, as this measure has been considered to more strongly reflect depression rather than other consequences of BD and misses large areas of mental HRQoL [64]. Our results obtained in a sample of euthymic participants suggest the opposite: the EQ-5D-3L index score was independently influenced by anxiety and functioning and, to a much lower extent, by residual depression. The classification of Grunze and Born [58] considered the possible impact of cognition on QoL in BD. Still, our results support neither a direct nor a total association between these two variables. In this classification, subsyndromal depression was considered a moderate correlate of QoL in BD, which was not supported by our study, perhaps due to the stringency of the criterion used to include euthymic participants. Finally, this classification reported a history of psychosis as having an unknown impact on QoL, whereas our results suggest a small albeit significant positive association between these two variables.

Several modifiable factors of psychosocial functioning were also identified in our study. Functioning was mainly influenced by subsyndromal depression, even though this variable showed a low range due to the stringent criterion used for inclusion. This finding is in accordance with previous cross-sectional [65] and longitudinal studies [16]. Our results suggest that targeting residual depressive symptoms, for instance, with adjunctive medication [66] and therapy programs [67–69], would improve functioning and QoL in BD at euthymia. The two other important correlates of functioning were cognition and anxiety. Although numerous studies showed a consistent association between cognition and functioning in BD (see, for example [70]), they are much more scarce concerning the association between anxiety and functioning (see [19] for a review). These results suggest tackling cognitive deficits with cognitive remediation programs [55, 56] would improve functioning in BD at euthymia. The history of psychosis was unrelated to functioning, thus confirming a previous finding that this specifier explains very little of the variance in functioning [71].

Finally, the model presented in this study revealed few modifiable factors for cognition, except the prescription of antipsychotics, which was mildly associated with poorer cognitive performance, and anxiety, which was weakly associated with it. A previous individual patient data meta-analysis reported that the use of antipsychotics was associated with specific measures of verbal memory [23]. However, it is impossible to draw any conclusions concerning a causal link between antipsychotics and cognitive impairment. Longitudinal studies are needed to clarify the effect of antipsychotics on cognition in BD. They should consider each molecule’s specific psychopharmacological properties, serum level, duration of exposure, and therapeutic response. Our results support a small negative impact of the history of psychosis on cognition in BD. Previous reports showed that cognitive impairments were associated with recent [72] and active psychotic features in BD but not with a history of remitted psychotic symptoms [73].

The present results must be interpreted in light of several limitations. First, the neuropsychological battery did not include measures of attention. Previous studies have identified attention as an important cognitive factor of the cognitive structure in BD through various factor analyses [46, 48]. In addition, our study lacked records of several important variables for QoL: medical and personality comorbidities, internalized stigma [74], and circadian rhythms, which have also been shown to be associated with QoL in BD [75]. There is little consensus within the literature on measuring QoL most accurately. Still, it may be argued that a measure of QoL specifically designed for BD [76] would have been preferable. Finally, the design of this study had two important limitations: the lack of comparison with a control group, which is required to infer whether the relationships presented in this model are specific to BD, and the cross-sectional design of this study, which makes it impossible to infer the direction of causality. The choice of the arrow direction from functioning to QoL was questionable, as some authors have postulated that psychological functioning measured with the Personal and Social Performance Scale may be influenced by perceived QoL in BD [11]. Longitudinal studies are needed to test the direction of the association between functioning and QoL, using, for instance, cross-lagged panel modeling, dual change score model, or latent difference score.

## Supporting information

Roux et al. supplementary material 1 Roux et al. supplementary material

Roux et al. supplementary material 2 Roux et al. supplementary material

Roux et al. supplementary material 3 Roux et al. supplementary material

## Data Availability

Due to ethical and legal restrictions, data involving clinical participants cannot be made publicly available. All relevant data are available upon request to the Fondation FondaMental for researchers who meet the criteria for access to confidential data.

## Supplementary material

The supplementary material for this article can be found at http://doi.org/10.1192/j.eurpsy.2024.1789.
