# Supplementary material for: Relationships between cognition, functioning, and quality of life of euthymic patients with bipolar disorder: Structural equation modeling with the FACE-BD cohort
Source: Eur Psychiatry. 2024 Nov 15;67(1):e78. doi: 10.1192/j.eurpsy.2024.1789 (PMC11730061; doi:10.1192/j.eurpsy.2024.1789)
Supplement: Roux et al. supplementary material 1 — Roux et al. supplementary material [file S0924933824017899sup001.pdf]

Supplementary Figure 1. Mediation model.
Covariates were omitted for readability (see Figure 2 for the model with covariates).

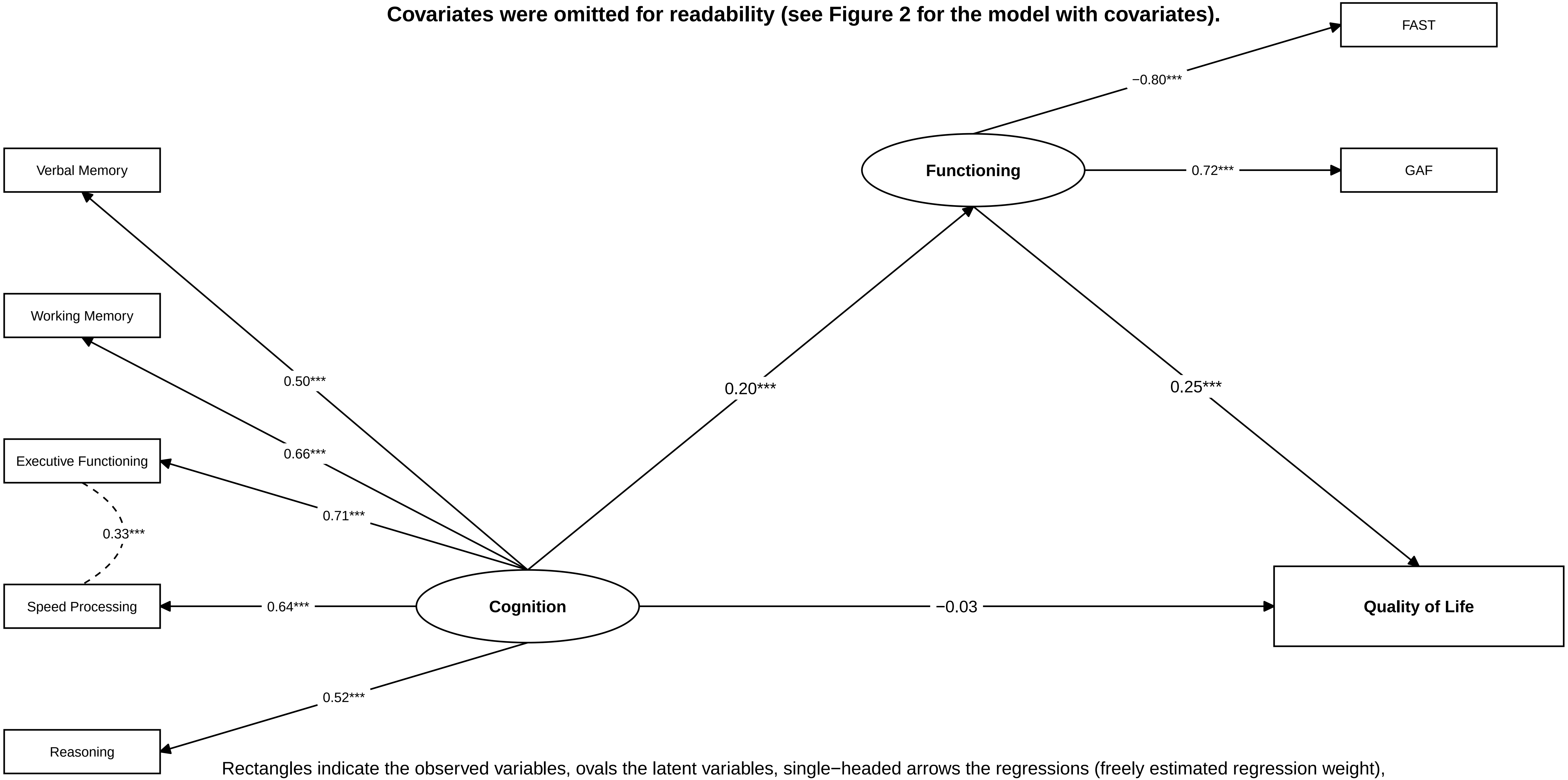

Rectangles indicate the observed variables, ovals the latent variables, single−headed arrows the regressions (freely estimated regression weight), double−headed arrows the covariances. Path coefficients were standardized. Significance levels are as follows: *** $P < 0.001$, ** $P < 0.01$, * $P < 0.05$.
FAST: Functioning Assesment Short Test; GAF: Global Assesment of Functioning.
