# Supplementary material for: Relationships between cognition, functioning, and quality of life of euthymic patients with bipolar disorder: Structural equation modeling with the FACE-BD cohort
Source: Eur Psychiatry. 2024 Nov 15;67(1):e78. doi: 10.1192/j.eurpsy.2024.1789 (PMC11730061; doi:10.1192/j.eurpsy.2024.1789)
Supplement: Roux et al. supplementary material 2 — Roux et al. supplementary material [file S0924933824017899sup002.docx]

**Supplementary Table 1**. Matrix of correlations between the variables included in the model

|  | **Verbal memory** | **Working memory** | **Executive functioning** | **Speed processing** | **Reasoning** | **GAF** | **FAST** | **EQ-5D** | **Antipsychotic** | **Psychotic features** | **MADRS** |
| --- | --- | --- | --- | --- | --- | --- | --- | --- | --- | --- | --- |
| Working memory | 0.28*** |  |  |  |  |  |  |  |  |  |  |
| Executive functioning | 0.32*** | 0.46*** |  |  |  |  |  |  |  |  |  |
| Speed processing | 0.33*** | 0.41*** | 0.62*** |  |  |  |  |  |  |  |  |
| Reasoning | 0.27*** | 0.36*** | 0.34*** | 0.28*** |  |  |  |  |  |  |  |
| GAF | 0 | 0 | 0.12*** | 0.12*** | 0 |  |  |  |  |  |  |
| FAST | -0.13*** | -0.16*** | -0.18*** | -0.15*** | -0.12*** | -0.55*** |  |  |  |  |  |
| EQ-5D-3L | 0 | 0 | 0 | 0 | 0 | 0.33*** | -0.34*** |  |  |  |  |
| Antipsychotic | -0.17*** | -0.14*** | -0.19*** | -0.17*** | 0 | 0 | 0.1** | 0 |  |  |  |
| Psychotic features | -0.12*** | 0 | -0.13*** | -0.18*** | 0 | 0 | 0 | 0.1** | 0.21*** |  |  |
| MADRS | 0 | 0* | 0 | -0.1** | 0 | -0.39*** | 0.41*** | -0.41*** | 0 | 0 |  |
| STAI-YA (state subscale) | 0 | -0.11*** | 0 | 0* | 0 | -0.33*** | 0.36*** | -0.54*** | 0 | 0* | 0.46*** |

***p < 0.001, **0.001 < p < 0.01, *0.01 < p < 0.05

GAF: Global Assessment of Functioning

FAST: Functioning Assessment Short Test

EQ-5D: European Quality of Life 5 dimensions and 3 lines

MADRS: Montgomery-Asberg Depression Rating Scale

STAI-YA: State-Trait Anxiety Inventory, form Y-A
