# Supplementary material for: Relationships between cognition, functioning, and quality of life of euthymic patients with bipolar disorder: Structural equation modeling with the FACE-BD cohort
Source: Eur Psychiatry. 2024 Nov 15;67(1):e78. doi: 10.1192/j.eurpsy.2024.1789 (PMC11730061; doi:10.1192/j.eurpsy.2024.1789)
Supplement: Roux et al. supplementary material 3 — Roux et al. supplementary material [file S0924933824017899sup003.docx]

**Supplementary Table 2.** Covariance coverage matrix

|  | **Verbal Memory** | **Working Memory** | **Executive Functioning** | **Processing speed** | **Reasoning** | **GAF** | **FAST** | **EQ-5D** | **MADRS** | **STAI-YA (state subscale)** | **Antipsychotics** | **Psychotic features** |
| --- | --- | --- | --- | --- | --- | --- | --- | --- | --- | --- | --- | --- |
| **Verbal Memory** | 0.81 | 0.8 | 0.81 | 0.81 | 0.79 | 0.7 | 0.79 | 0.65 | 0.8 | 0.77 | 0.65 | 0.66 |
| **Working Memory** | 0.8 | 0.82 | 0.82 | 0.82 | 0.81 | 0.72 | 0.81 | 0.67 | 0.81 | 0.79 | 0.66 | 0.68 |
| **Executive Functioning** | 0.81 | 0.82 | 0.82 | 0.82 | 0.81 | 0.72 | 0.81 | 0.67 | 0.81 | 0.79 | 0.66 | 0.68 |
| **Processing speed** | 0.81 | 0.82 | 0.82 | 0.83 | 0.81 | 0.72 | 0.81 | 0.67 | 0.82 | 0.79 | 0.66 | 0.68 |
| **Reasoning** | 0.79 | 0.81 | 0.81 | 0.81 | 0.81 | 0.71 | 0.8 | 0.66 | 0.8 | 0.78 | 0.65 | 0.67 |
| **GAF** | 0.7 | 0.72 | 0.72 | 0.72 | 0.71 | 0.85 | 0.85 | 0.69 | 0.85 | 0.82 | 0.68 | 0.72 |
| **FAST** | 0.79 | 0.81 | 0.81 | 0.81 | 0.8 | 0.85 | 0.97 | 0.79 | 0.97 | 0.92 | 0.76 | 0.81 |
| **EQ-5D** | 0.65 | 0.67 | 0.67 | 0.67 | 0.66 | 0.69 | 0.79 | 0.8 | 0.79 | 0.79 | 0.62 | 0.66 |
| **MADRS** | 0.8 | 0.81 | 0.81 | 0.82 | 0.8 | 0.85 | 0.97 | 0.79 | 0.98 | 0.93 | 0.76 | 0.81 |
| **STAI-YA (state subscale)** | 0.77 | 0.79 | 0.79 | 0.79 | 0.78 | 0.82 | 0.92 | 0.79 | 0.93 | 0.94 | 0.74 | 0.78 |
| **Antipsychotics** | 0.65 | 0.66 | 0.66 | 0.66 | 0.65 | 0.68 | 0.76 | 0.62 | 0.76 | 0.74 | 0.78 | 0.65 |
| **Psychotic features** | 0.66 | 0.68 | 0.68 | 0.68 | 0.67 | 0.72 | 0.81 | 0.66 | 0.81 | 0.78 | 0.65 | 0.82 |

GAF: Global Assessment of Functioning

FAST: Functioning Assessment Short Test

EQ-5D: European Quality of Life 5 dimensions and 3 lines

MADRS: Montgomery-Asberg Depression Rating Scale

STAI-YA: State-Trait Anxiety Inventory, form Y-A
